# Research on the Compression Algorithm of the Infrared Thermal Image Sequence Based on Differential Evolution and Double Exponential Decay Model

**DOI:** 10.1155/2014/601506

**Published:** 2014-02-13

**Authors:** Jin-Yu Zhang, Xiang-Bing Meng, Wei Xu, Wei Zhang, Yong Zhang

**Affiliations:** ^1^Xi'an Research Institute of High-Tech, 2 Tongxin Road, Xi'an, Shaanxi 710025, China; ^2^Keppel Offshore & Marine Technology Centre Pte Ltd, 31 Shipyard Road, Singapore 628130

## Abstract

This paper has proposed a new thermal wave image sequence compression algorithm by combining double exponential decay fitting model and differential evolution algorithm. This study benchmarked fitting compression results and precision of the proposed method was benchmarked to that of the traditional methods via experiment; it investigated the fitting compression performance under the long time series and improved model and validated the algorithm by practical thermal image sequence compression and reconstruction. The results show that the proposed algorithm is a fast and highly precise infrared image data processing method.

## 1. Introduction

In recent years, infrared thermal wave nondestructive testing technology as an emerging means of nondestructive testing has drawn wide research interest. It has been applied in aerospace, power generation, material, medicine, construction, and other nondestructive testing fields. However, the large amount of information of the thermographic sequence generated during the detection has seriously restricted the image processing and storage. To compress and reconstruct the thermographic sequence efficiently and accurately is the basis of postprocessing and recognition of the thermal graphs, which is also the key to quickly and accurately detect the defects [1–5].

Researchers have proposed a number of processing methods to reduce noise and enhance the contrast ratio of defects. The renowned ones are differentiation method, polynomial fitting method [1, 2], regularization method, PCA method [3], correlation coefficient method [4], pulsed phase method [5], and so forth. All these methods employ certain fitting model and compression algorithm of thermographic sequences.

Polynomial fitting is one of the traditional and typical fitting models. Shepard et al. did a lot of studies on polynomial fitting and proposed a unique thermographic sequence reconstruction (TSR) method [6]. His proposed method firstly took the logarithm of the original data; then it used polynomial fitting model to fit the processed data and the model parameters to achieve image compression. It achieved good results in practical applications. However, since polynomial fitting model uses the least squares method, it may fall into local minima rather than the global one in the solution searching process [7, 8]. And for handling nonlinear data fitting problems, the least squares method could only approximate it into a linear problem by taking the logarithm of both ends which resulted in migration of the optimal solution [9].

Zhang et al., through their in-depth research of the thermal wave transmission mechanism, proposed the nonlinear *L*-*M* fitting method based on the thermal theoretical model. This method aimed to overcome the shortcoming of polynomial fitting and it achieved good results [10]. In order to resolve problems presented in polynomial fitting model, Zhang et al. proposed a thermographic sequences fitting algorithm based on genetic algorithm (GA) which is more competent in searching for global optimal solution and gives more accurate processing results [11]. But both methods need a large amount of computation and long processing time; this makes them unable to meet the actual engineering needs.

In practice, it is found that the nonlinear fitting method based on theoretical model has the problem of being inaccurate since the temperature change of thermal image follows double exponential decay model. When using double exponential model for fitting, we usually use Gauss-Newton method, conjugate gradient method, or damped least squares method. However, these methods highly depend on the selection of initial conditions. They have difficulty in searching for global optimal solution. The convergence is slow, or may even diverge. To solve these problems, some scholars proposed some improved methods. Chen et al. proposed a homotopy alternating iterative method which effectively reduced the level of dependence of initial condition and made large-scale convergence possible. But the dependence on initial conditions still require attention [12].

Differential evolution (DE) was proposed by Storn and Price in 1995, mainly for the optimization problems of real number [13–15]. The algorithm is a self-adaptive community-based global optimization algorithm and it belongs to the evolutionary algorithm family. It has characteristics such as simple structure, easy implementation, fast convergence, and strong robustness.

To take the advantages of double exponential decay model and differential evolution algorithm, this paper combined them into a new set of thermal image data fitting and compression algorithm. This algorithm not only can overcome the problems of polynomial fitting to improve the accuracy of fitting but also can improve computational efficiency and robustness.

The rest of that content of this paper is organized as follows.  Section 1 is the introduction of DE and double decay model; the new algorithm is described in Section 2; Section 3 introduces the experiment and analysis of experimental results; the last section is the summary.

## 2. Principle of Algorithm 

### 2.1. Principle of DE Algorithm

Differential evolution as a kind of evolutionary algorithm has similar process with other evolutionary algorithms. DE needs population initialization, individual fitness evaluation, group evolution, and so forth [13]. Starting from a randomly generated initial population, new individuals are produced by summing the vector difference between any two individuals in the population to the third individual. Then the new individual is compared to the corresponding individual in the contemporary population. If the fitness of the new individual is better than that of the corresponding individual, it will be replaced with the new one in the next generation. Otherwise, the old individual will be preserved. Through continuing iterative evolutions, retaining superior individuals are retained and inferior ones are weeded out. DE algorithm guides the searching to approach the optimal solution. The evolutionary process is as follows [13–16].

1. Determine control parameters of the DE algorithm and the fitness function. The control parameters include population size (NP), scaling factor (*F*), and crossover probability (CR).
2. Randomly generate initial population.
3. Evaluate the initial population, that is, to calculate the fitness value of each individual in the initial population.
4. Judge whether either the condition of termination or the maximum evolutionary generation number has been reached. If yes, stop the evolution and output the best individual as the optimal solution; if not, continue.
5. Conduct mutation and crossover operation to obtain an intermediate population.
6. Select individuals among the original population and the intermediate population to obtain a new population.
7. Increment the evolution generation count *g* = *g* + 1, then go to step (4).

The detailed operation is interpreted as follows.

(*1) Generate the Initial Population.* Initial population can be expressed as {*x*_*i*_(0) | *x*_*j*,*i*_^min⁡^ ≤ *x*_*j*,*i*_(0) ≤ *x*_*j*,*i*_^max⁡^; *i* = 1,2,…, NP; *j* = 1,2,…, *D*}.

Each individual is generated using the following formula:

(1) $$\begin{array}{c} x_{j,i}\left(0\right)=x_{j,i}^{\min}+\text{rand}\left(0,1\right)\cdot \left(x_{j,i}^{\max}-x_{j,i}^{\min}\right). \end{array}$$

In formula (1), NP represents the number of individuals in the population and *x*_*j*,*i*_(0) represents the *j*th dimension component of the *i*th individual in the initial population; rand(0,1) represents the random function, and the value is within the range [0,1]. 

(*2) Mutation Operation.* Individual mutation in DE algorithm is achieved through selected differential mutation strategy. The basic differential mutation strategy is to randomly select two distinct individuals in the population; then summing the weighted vector difference of the two individuals to the current individual, the formula is expressed as

(2) $$\begin{array}{c} \text{DE}/\text{rand}/1:\ldots v_{i}\left(g+1\right)=x_{r1}\left(g\right)+F\cdot \left[x_{r2}\left(g\right)-x_{r3}\left(g\right)\right]. \end{array}$$

In formula (2), *i* ≠ *r*1 ≠ *r*2 ≠ *r*3, *F* is the scale factor, and *x*_*i*_(*g*) represents the *i*th individual of the *g*th generation population.

Besides the basic mutation strategy, DE researchers also designed other mutation strategies [11, 12]. 

(*3) Crossover Operation.* Crossover operation is conducted on the intermediate individual *x*_*i*_(*g* + 1) obtained from mutation:

(3) $$\begin{array}{c} u_{j,i}\left(g+1\right)=\{\begin{array}{cc} u_{j,i}\left(g+1\right) & \text{if}\;\;(\text{rand}\left(0,1\right)\leq \text{CR}\;\text{or}\;\;j=j_{\text{rand}}),\\ x_{j,i}\left(g\right) & \text{otherwise}. \end{array} \end{array}$$

In formula (3), CR is crossover probability, and *j*_rand_ is a random integer within the interval [1, *D*]. This crossover strategy can ensure that individual *u*_*j*,*i*_(*g* + 1) contains the content of individual *u*_*j*,*i*_(*g*). 

(*4) Selection Operation.* Child individuals are generated after mutation and crossover operation; then the child individual is compared with the corresponding parent individual in a one-to-one select way. Superior individual is selected according to the fitness value into the next generation population. The selection operator can be expressed as follows:

(4) $$\begin{array}{c} x_{i}\left(g+1\right)=\{\begin{array}{cc} u_{i}\left(g+1\right) & \text{if}\;f\left(u_{i}\left(g+1\right)\right)\leq f\left(x_{i}\left(g\right)\right),\\ x_{i}\left(g\right) & \text{otherwise}, \end{array} \end{array}$$

where *f*(*x*_*i*_(*g*)) is the fitness value of individual *x*_*i*_(*g*).

### 2.2. Thermal Image Double Exponential Decay Model

Double exponential decay model can effectively describe physical processes such as the smooth lightning full waveform, the induced polarization potential of polarization potential logging, and high-altitude nuclear electromagnetic pulse waveform. It also has been well applied in other areas [17–21].

Typical pulsed thermographic NDE signal sequences are composed of *N* consecutive frames of two-dimensional thermal images, whose sampling rate is 1/Δ*t* (Δ*t* is the time interval between two thermal images). If each frame has *L* × *M* elements (or pixels), the entire sequence is an *L* × *M* × *N* three-dimensional array of thermal response value. The corresponding temperature value (or thermal radiation intensity value) of each thermal image pixel {(*i*, *j*) | *i* = 1,2,…, *L*; *j* = 1,2,…, *M*} can be seen as the function of temperature with time after flash excitation. This function can be described with a double exponential decay model.

Given that the time is *x*_*i*_ and the corresponding temperature value of a pixel is *y*_*i*_, the coefficients of the mathematical model *y* = *ae* ^−*bx*^ + *ce* ^−*dx*^ are *a*, *b*, *c*, and *d*. Then the fitting problem of the function can be transformed into the following unconstrained nonlinear optimization problem as follows:

(5) $$\begin{array}{c} \min\sum_{i=1}^{n}{\left(ae^{-bx_{i}}+ce^{-dx_{i}}-y_{i}\right)}^{2}. \end{array}$$

In some practical applications, *a*, *b*, *c*, and *d* are requested to be nonnegative. This problem can be transformed into a constrained nonlinear optimization problem as follows:

(6) $$\begin{array}{c} \min\sum_{i=1}^{n}{\left(ae^{-bx_{i}}+ce^{-dx_{i}}-y_{i}\right)}^{2}\;\left(a,b,c,d\geq 0\right). \end{array}$$

## 3. Thermal Image Fitting Compression Algorithm

In practice, it is found that using double exponential decay model to fit the data of infrared thermal wave images can achieve better fitting effects as it can suppress noise. Meanwhile, since there are only four fitting coefficients, fewer than six coefficients of TSR method, the fitting coefficients can be used to replace a specific pixel's time sequence whose length is *N* (generally 200 to 2000). This gives a compression ratio of about 50 to 500. Obviously, this can greatly reduce the thermal image storage space.

**Figure 1:**
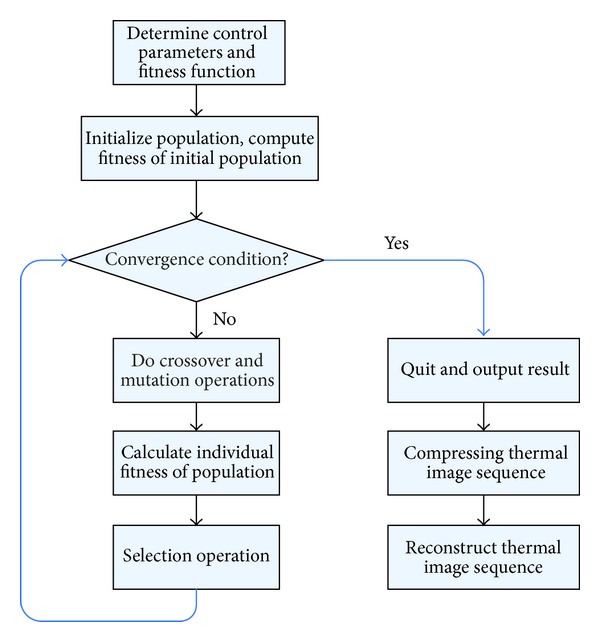
Compressing flowchart of thermal image sequence.

When combined with the DE algorithm which eliminates the dependence on initial value, not only global convergence is achieved but also fitting speed has been greatly improved. Therefore, this paper proposes a new infrared thermal image compression method based on double exponential decay model and DE algorithm. The flow chart of proposed method is shown in Figure 1, and the detailed implementation process is as follows.

1. Determine the DE control parameters, namely, population size NP, crossover probability CR, the scaling factor *F*, and set the maximum iteration number Gm, and convergence decision condition.
2. Based on double exponential decay model, a fitness function = ∑_*i*=1_^*n*^(*ae* ^−*bx*_*i*_^+*ce* ^−*dx*_*i*_^−*y*_*i*_)^2^ is built. The data set {(*x*_*i*_, *y*_*i*_) | *i* = 1,…, *N*} is the corresponding thermal graphic sequence of a specific pixel.
3. Randomly generate initial population, and set the dimension of each individual in the population to be four according to the fitness function.
4. Calculate the initial population fitness value of each individual.
5. Determine whether either convergence condition is met or the maximum evolution generation is reached. If yes, terminate the evolution and go to step (9); if not, continue to next step.
6. Execute mutation and crossover operations to obtain an intermediate population by randomly selecting corresponding number of individuals from the previous generation population (the first generation is the initial population) based on the selected mutation strategy.
7. Calculate fitness value of each individual in the initial population and the intermediate population and compare them one by one; then select the individual with smaller fitness value in each group of individuals to form a new generation population.
8. Increment the generation count *g* = *g* + 1, go to step (5).
9. Select the individual with the minimum fitness value when evolution converges or reaches the maximum evolution generation number. The values of double exponential model parameter, *a*, *b*, *c*, and *d*, are the only data to be compressed and stored for the pixel.
10. Repeat step (1) to (9) for all pixels of the thermal image.
11. Retrieve and fill parameters *a*, *b*, *c* and *d* into fitting model *f*(*x*_*i*_) = *ae* ^−*bx*_*i*_^ + *ce* ^−*dx*_*i*_^ to reconstruct time sequence *f*(*x*_*i*_) with the known time value of *x*_*i*_.

## 4. Experimental Results and Analysis

In nondestructive testing, in order to assess the performance of the testing method and system in a more accurate and visible way, generally an embedded defective test specimen will be used to test the effectiveness of the inspection method. In this study, we used an active infrared thermal wave imaging device to test a specimen made of steel.

The thermal Imager is VarioCAM hr research 680 thermal imager from InfraTec company. Its spatial resolution is 640∗480, the maximum frame rate is 60 Hz, infrared spectral response range is 7.5~14 um, and imaging rate is 50 Hz. We used pulse heating single side positioning detection method: the heat source is two high-power flash lamps, the heating power range is 0~4.8 KJ, the distance between the inspection position and the test specimen is about 500 mm, and the heating pulse lasting duration is about 2 ms.

**Figure 2:**
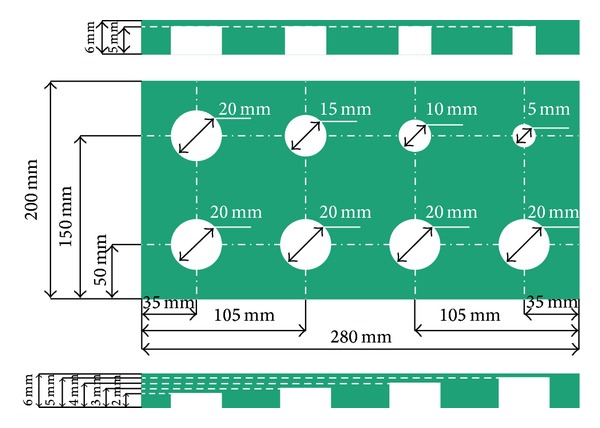
Defect distribution and depth.

**Figure 3:**
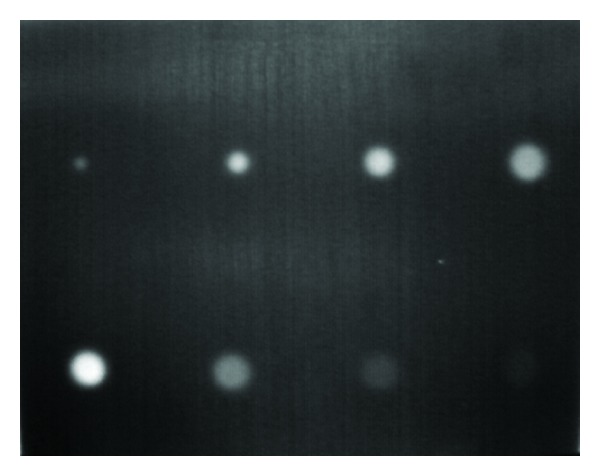
Single frame thermal image of steel shell specimen.

Test specimen's length is 280 mm, width is 200 mm, and thickness is 6 mm. There are eight flat bottom holes at the back of the specimen to simulate debonding defect; the four holes on top have the same depth of 1 mm and diameters of 20 mm, 16 mm, 10 mm, and 5 mm, respectively; the four holes below are with the same diameter of 20 mm and depths of 2 mm, 3 mm, 4 mm, and 5 mm, respectively. The dimension and location of the holes are shown in Figure 2 and a single frame thermal image of the specimen is shown in Figure 3.

### 4.1. Fitting Compression of Single Point's Short Thermal Graphic Sequence

**Figure 4:**
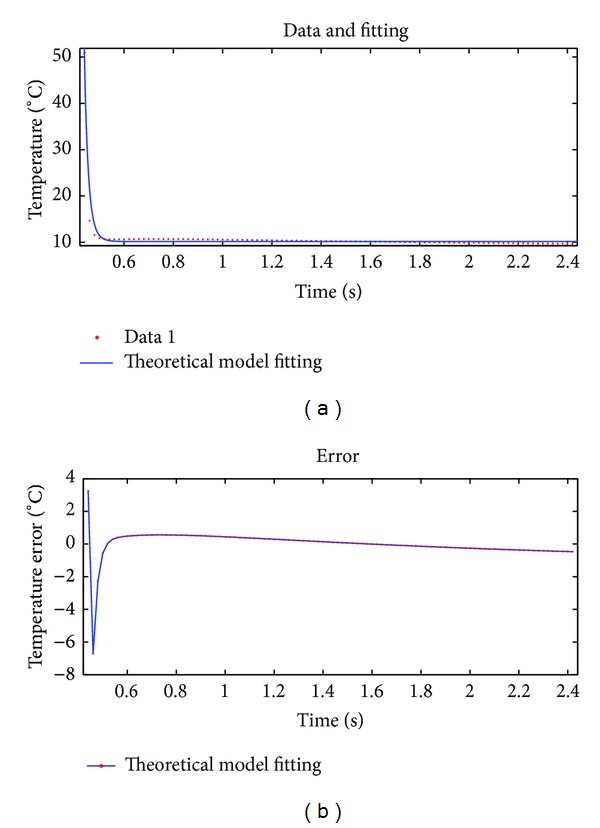
Result of fitting data 1 by using theoretical model.

**Figure 5:**
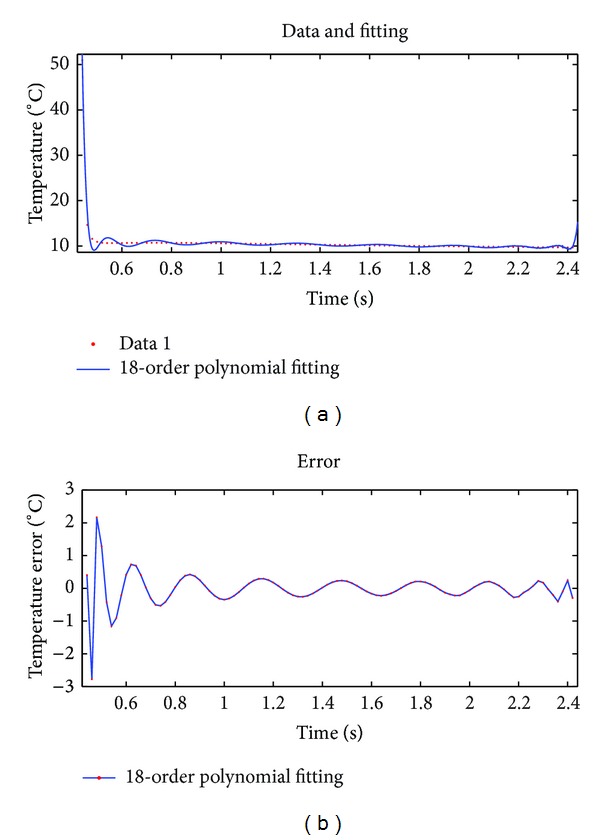
Fitting results using 18-order polynomial.

**Figure 6:**
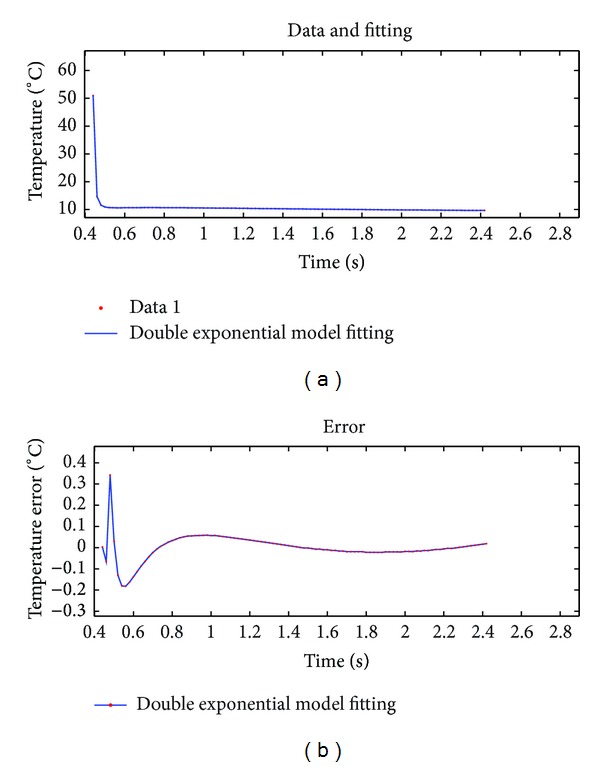
Fitting results using double exponential model.

We selected a representative point (at the center of a defective area) and used its thermal graphic sequence for this experiment. Three methods, namely, theoretical models, 18-order polynomial, and double exponential decay model, were used to fit the same set of data. Results by using three methods are compared. Quantitative analysis of the results of three methods is performed through comparing their fitting effect evaluation parameter values. The fitting results of three methods were shown in Figures 4–6.

**Figure 7:**
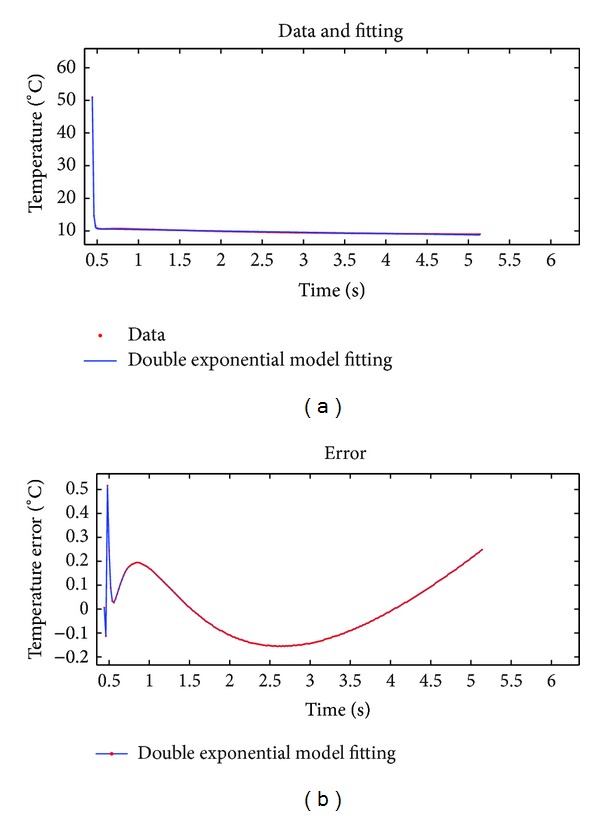
Results of fitting 250-frame thermal image data by using proposed algorithm.

**Figure 8:**
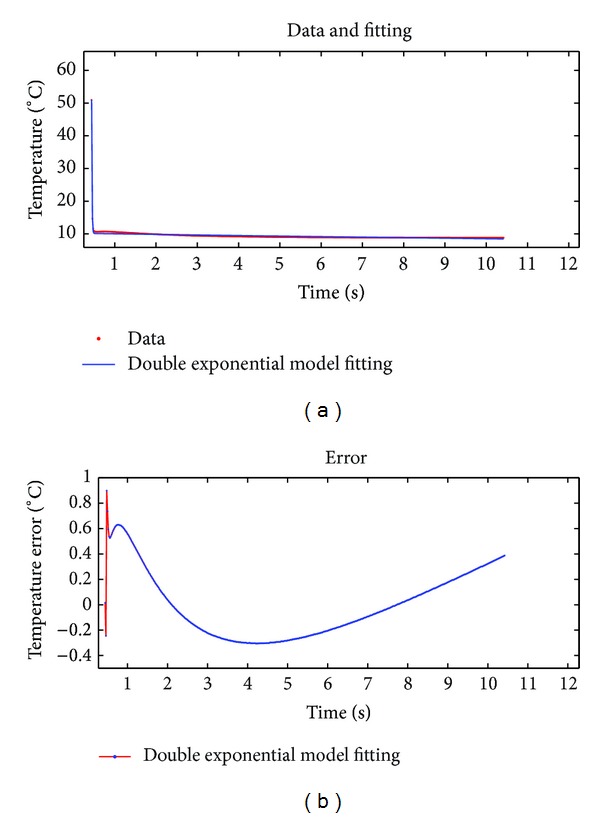
Results of fitting 500-frame thermal image data by using proposed algorithm.

**Figure 9:**
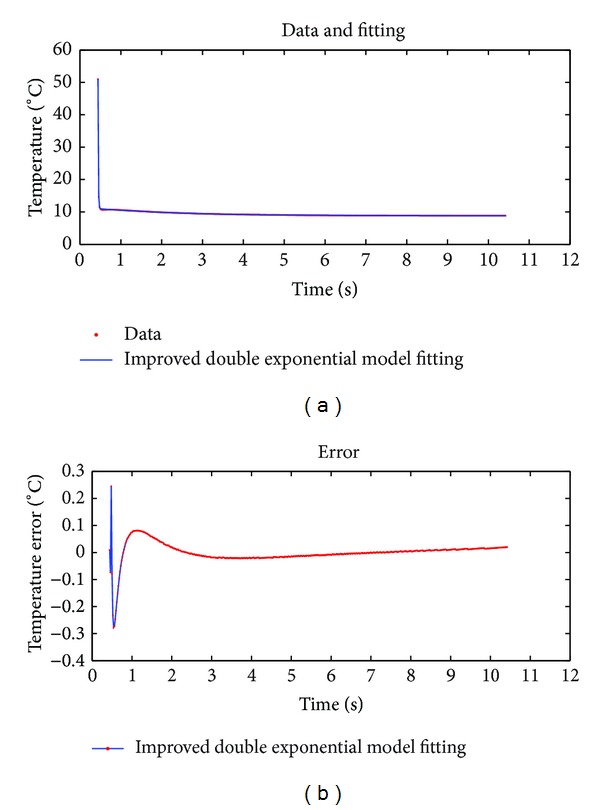
Result of fitting 500-frame thermal image data by using the improved model.

Note that Figures 4, 5, and 6 are the fitting results of theoretical model, 18-order polynomial, and double exponential decay model, respectively. The two curves on top of each figure are the data sequence curve and corresponding fitting curve, respectively. The curve below is the fitting error curve. And Figures 7, 8, and 9 have the same layout.

Fitting effect evaluation parameters of three methods are shown in Table 1.

According to Figures 4–6 and Table 1, for the same set of experimental data, among three fitting methods, accuracy of double exponential model based on differential evolution algorithm is significantly higher than that of the other two methods, about 63 times in max error, and this model uses only four parameters, which demonstrates a superior compression ratio of data.

### 4.2. Fitting Compression of Single-Point's Long Thermal Graphic Sequence

For long image sequence, double exponential model is also able to achieve a high fitting accuracy, which cannot be achieved by polynomial fitting method or theoretical models. We used the same test specimen and inspection equipment to capture 250 and 500 frames of thermal images, respectively, then took the thermal graphic sequence of a specific point. We used our proposed method to fit two sets of data. The selected DE algorithm mutation strategy is DE/best/1 : *v*_*i*_ = *x*_best_ + *F* · (*x*_*r*2_ − *x*_*r*3_), the population size NP is 40, the crossover probability CR is 0.9, the scaling factor *F* is 0.5, and the maximum number of iterations is 1000. The fitting result is shown in Figures 7 and 8.

Fitting effect evaluation parameters of the two sets of experimental data are shown in Table 2.

Figures 7 and 8 and Table 2 show that the new fitting method can maintain a high fitting precision level when fitting long thermal graphic sequences. The error is relatively small. The fitting speed is very fast, and the fitting curves are relatively stable without fluctuating phenomenon, while theoretical model fitting and polynomial fitting can not achieve this effect [10, 11]. Therefore, the fitting compression method proposed in this paper is an efficient method for processing infrared thermal image data.

### 4.3. Fitting Compression of Double Exponential Model with Constant Term

During the experiment, we also found that a double exponential decay model with a constant term *h*, which is a correction parameter, can further improve the fitting accuracy with remarkably reduced fitting error. The model equation is rewritten as *y* = *ae* ^−*bx*^ + *ce* ^−*dx*^ + *h*.  Figure 9 shows the results of fitting aforementioned 500-frame thermal graphic sequence by using the double exponential model with correction term.  Table 3 shows the fitting effect evaluation parameters by using the two double exponential models with or without constant term.

The fitting effect evaluation parameter values of the two models are shown in Table 3.

By comparing the fitting effect evaluation parameter values in Figures 8 and 9 and Table 3, it can be seen that the fitting accuracy has been significantly improved when a constant term as a correction parameter is added to double exponential model to fit the infrared thermal graphic sequence. However, one more fitting compressing parameter is added to the model.

### 4.4. Compression and Reconstruction of the Whole Single Frame Thermal Image

**Figure 10:**
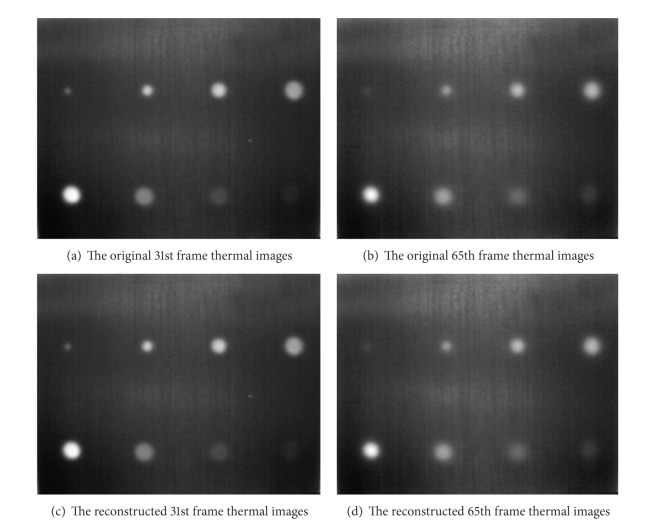
Original thermal images and reconstructed thermal images.

Figure 10 shows the comparison of the original thermal images to the reconstructed thermal images processed by our proposed method. Figures 10(a) and 10(b) are the original 31st and 65th frame thermal images, respectively. Figures 10(c) and 10(d) are the reconstructed 31st and 65th frame thermal images, respectively. Two pairs of corresponding frame images are almost exactly the same without any difference that can be identified intuitively. Their square error is almost 0. Thus, it can be seen that this method can meet the practical engineering needs completely.

Note that, from Tables 1 to 3, it can be seen that the overall error of this new fitting method is very small. And from Figures 4 to 9, the main fitting errors appear in the first few frames. In addition, the thermal imager used in experiments has good noise resistance and digitalization of thermal image capturing, so there is almost no error between the original thermal images and the reconstructed ones specific to the 31st and 65th frames in Figure 10.

In summary, the fitting compression method based on differential evolution algorithm and double exponential decay model is an optimum infrared thermal wave image processing method with high accuracy and precision, high compression ratio, high processing speed. It has a high commercial value and prospective engineering application.

## 5. Conclusions

We have fully discussed the image fitting compressing algorithm of pulsed thermal wave image sequence based on the combination of DE algorithm and double exponential decay model. And we also compared the fitting and compressing effect of this method to that of the traditional method with real experimental data, studied the fitting and compressing performance using short and long time sequence, investigated an improved model, and validated the effect of the method by compressing and reconstructing the whole single frame thermal image.

The results show that there are significant advantages of this new fitting and compressing method over polynomial fitting method and the TSR method. The fitting speed is fast because it contains fewer compressing parameters. Its data compression ratio is high, especially in fitting long thermal graphic sequence. Compared with the traditional optimization algorithms, this method is not affected by initial value, and it has good global convergence with a remarkably improved convergence rate. It can also achieve high fitting accuracy. All these serve the needs of demanding engineering applications well; thus, it will be an ideal fitting and compressing method for infrared thermal image sequence.

## Figures and Tables

**Table 1:** Fitting effect of theoretical model, 18-order polynomial, and double exponential model.

Evaluation parameters	Theoretical model	18-order polynomial	Double exponential model
SSE	24.64	22.01	0.35
*R*-square	0.9852	0.9868	0.9999
Adjusted *R*-square	0.9849	0.9839	0.9997
RMSE	0.504	0.5213	0.0588

SSE: the sum of squares due to error; *R*-square: coefficient of determination; adjusted *R*-square: degree-of-freedom adjusted coefficient of determination; RMSE: root means squared error.

**Table 2:** Fitting effect evaluation parameters of thermal image data with 250 and 500 frames.

Evaluation parameters	250 frames	500 frames
SSE	3.86	35.27
*R*-square	0.9989	0.9907
Adjusted *R*-square	0.9978	0.9815
RMSE	0.1279	0.2656

**Table 3:** Effect of evaluation parameter values of 500-frame thermal image data fitted by model with or without constant term.

Evaluation parameters	Initial model (w/o constant term)	Improved model (w constant term)
SSE	35.27	0.89
*R*-square	0.9907	0.9998
Adjusted *R*-square	0.9815	0.9995
RMSE	0.2656	0.0422

## References

[1] Ibarra-Castanedo C, Piau J, Guilbert S (2009). Comparative study of active thermography techniques for the nondestructive evaluation of honeycomb structures. *Research in Nondestructive Evaluation*.

[2] Ahn H, Choi C, Kim K (1993). Iterative learning control for a class of nonlinear systems. *Automatica*.

[3] Marinetti S, Grinzato E, Bison PG (2004). Statistical analysis of IR thermographic sequences by PCA. *Infrared Physics and Technology*.

[4] Guo X, Vavilov V, Guo G, Shao W, Liu Y (2004). Modeling and image processing in infrared thermographic NDT of composite materials. *Journal of Beijing University of Aeronautics and Astronautics*.

[5] Maldague X, Marinetti S (1996). Pulse phase infrared thermography. *Journal of Applied Physics*.

[6] Shepard SM, Ahmed T, Rubadeux BA, Wang D, Lhota JR (2001). Synthetic processing of pulsed thermographic data for inspection of turbine components. *Insight*.

[7] Maldague XPV, Moore PO (2001). *Nondestructive Testing Handbook Infrared and Thermal Testing*.

[8] Guo X, Shao W, Guo G, Liu Y (2005). Image processing algorithms for uneven heating in infrared thermographic NDT. *Journal of Beijing University of Aeronautics and Astronautics*.

[9] Zhang F (2007). A comparative analysis between the genetic algorithm and least squares method in data processing. *College Physics*.

[10] Zhang Y, Zhang J, Huang J (2012). Infrared thermal imaging data fitting method based on theoretical model of infrared thermal wave detection. *Infrared*.

[11] Zhang Y, Zhang J, Huang X, Huang J (2012). The infrared thermal imaging data fitting method based on genetic algorithm. *Nondestructive Testing*.

[12] Chen H, Deng S, Fan Y (2007). Application of homotopy alternative iteration method in double exponential fitting. *Computer Engineering and Applications*.

[13] Storn R, Price K (1995). Differential evolution-A simple and efficient adaptive scheme for global optimization over continuous spaces. *Technical Report*.

[14] Storn R, Price K (1997). Differential evolution—a simple and efficient heuristic for global optimization over continuous spaces. *Journal of Global Optimization*.

[15] Price K, Storn R, Lampinen J (2005). *Differential Evolution: A Practical Approach for Global Optimization*.

[16] Noman N, Iba H (2008). Accelerating differential evolution using an adaptive local search. *IEEE Transactions on Evolutionary Computation*.

[17] Hao Y, Wang G, Li Y (2000). Parameter extraction method of impact wave based on double exponential function fitting. *High Voltage Engineering*.

[18] Chen X, Huang K, Zhao X (2002). A study of nonlinear data-fitting based on a GA in electrical log. *Journal of Sichuan University*.

[19] Mao C, Guo X, Zhou H, Xie Y (2004). Fitting method of the simulated HEMP waveform by the double-exponential function. *High Power Laser and Particle Beams*.

[20] Han D, Jiang C, Fu P, Mou Y, Yang C (2010). Polynomial and bi-exponential curve fitting for discrete-time sequences of detector signals and its application in digitized nuclear spectrum. *Nuclear Electronics and Detection Technology*.

[21] Bian M, Li K (2005). Estimation of longitudinal road friction coefficient based on a dual-exponential model. *Transactions of the Chinese Society of Agricultural Machinery*.

